# Proof-of-Concept Study of Drug Brain Permeability Between *in Vivo* Human Brain and an *in Vitro* iPSCs-Human Blood-Brain Barrier Model

**DOI:** 10.1038/s41598-019-52213-6

**Published:** 2019-11-05

**Authors:** Gwenaëlle Le Roux, Rafika Jarray, Anne-Cécile Guyot, Serena Pavoni, Narciso Costa, Frédéric Théodoro, Ferid Nassor, Alain Pruvost, Nicolas Tournier, Yulia Kiyan, Oliver Langer, Frank Yates, Jean Philippe Deslys, Aloïse Mabondzo

**Affiliations:** 1 Service de Pharmacologie et d’Immunoanalyse, CEA, Université Paris-Saclay, F-91191 Gif-sur-Yvette, France; 2 Service d’Etude des Prions et des Infections Atypiques, CEA, F-92265 Fontenay-aux-Roses, France; 3 Sup’Biotech, F-94800 Villejuif, France; 4 Service de Pharmacologie et d’Immunoanalyse (SPI), Plateforme Smart-MS, CEA, INRA, Université Paris-Saclay, F-91191 Gif-sur-Yvette, France; 5 UMR 1023 IMIV, Service Hospitalier Frédéric Joliot, CEA, Inserm, Univ. Paris Sud, CNRS, Université Paris-Saclay, Orsay, France; 6 Medizinische Hochschule Hannover, DE-30625 Hannover, Germany; 7 Department of Clinical Pharmacology, Medical University of Vienna, A-1090 Vienna, Austria; 8 Preclinical Molecular Imaging, AIT Austrian Institute of Technology GmbH, A-2444 Seibersdorf, Austria

**Keywords:** Blood-brain barrier, Induced pluripotent stem cells

## Abstract

The development of effective central nervous system (CNS) drugs has been hampered by the lack of robust strategies to mimic the blood-brain barrier (BBB) and cerebrovascular impairments *in vitro*. Recent technological advancements in BBB modeling using induced pluripotent stem cells (iPSCs) allowed to overcome some of these obstacles, nonetheless the pertinence for their use in drug permeation study remains to be established. This mandatory information requires a cross comparison of *in vitro* and *in vivo* pharmacokinetic data in the same species to avoid failure in late clinical drug development. Here, we measured the BBB permeabilities of 8 clinical positron emission tomography (PET) radioligands with known pharmacokinetic parameters in human brain *in vivo* with a newly developed *in vitro* iPSC-based human BBB (iPSC-hBBB) model. Our findings showed a good correlation between *in vitro* and *in vivo* drug brain permeability (R^2^ = 0.83; *P* = 0.008) which contrasted with the limited correlation between *in vitro* apparent permeability for a set of 18 CNS/non-CNS compounds using the *in vitro* iPSCs-hBBB model and drug physicochemical properties. Our data suggest that the iPSC-hBBB model can be integrated in a flow scheme of CNS drug screening and potentially used to study species differences in BBB permeation.

## Introduction

For central nervous system (CNS) drugs, the step from *in vitro* activity to *in vivo* efficacy depends critically on the ability of the drug to cross the blood-brain barrier (BBB). The BBB is not merely a physical barrier but rather is an exquisitely modulated, functional gate^1^. Sensitive to biochemical signals from its local environment, it controls the influx and efflux of a wide variety of substances presumably required for optimal brain function^2^. The development of neuroactive substances in the pharmaceutical industry is an extremely costly endeavour plagued by a very high risk due to the high attrition rate in various stages of development. The success rate from first-in-human to registration for neuroactive drugs targeting the CNS is significantly lower than for other indications, such as cardiovascular, infectious, inflammatory, and metabolic diseases^3,4^. The development of drugs targeting the CNS requires precise knowledge of their penetration into the brain^2^, and ideally, this information should be obtained as early as possible to avoid failure in late clinical development, when upward of $100 million is typically invested in a drug candidate. Therefore, it is important to develop *in vitro* reliable screening tools that will allow the optimization of neuroactive molecules for brain penetration. Our group conceived and first developed static rodent and human BBB models using primary cells^5–8^. Due to the difficulties to get access to human brain tissue, several groups have attempted to develop *in vitro* human BBB models using induced pluripotent stem cells (iPSCs). Several *in vitro* models have been proposed^9,10^. These 2D/3D models are based on the use of iPSCs in static or microfluidic conditions^11–20^. Whatever their ressemblance to the *in vivo* BBB, *in vitro* BBB models must be carefully assessed for their ability to predict accurately the passage of drugs into the CNS *in vivo*. Nowadays, all models, whatether their characteristics are validated using the parameters of drug BBB passage measured directly in laboratory animals. Due to the poor predictive value of animal models for human responses to drugs, the cross-correlations between the *in vitro* human BBB model and the pharmacokinetic data in the *in vivo* human brain is mandatory to demonstrate the predictive value of the *in vitro* screening tools.

Here we report the development of a human BBB model using two different iPSC lines based on the optimization of the protocol previously reported^15^. Using these iPSC lines, we addressed their differentiation into brain endothelial cells (BECs) and their capacity to generate a tight monolayer in co-culture with glial cells. We also compared the permeability of 8 compounds using this *in vitro* iPSC-hBBB model with quantitative clinical data regarding their BBB permation obtained using brain PET imaging. We found a good correlation between the *in vitro* and *in vivo* drug brain permeability (R^2^ = 0.83; *P* = 0.008). In addition, we noted a lack of relationship between *in vitro* apparent permeability for 18 CNS and non-CNS compounds using the *in vitro* iPSC-hBBB model and the compounds’ physicochemical properties. Even if the BBB permeabilities showed the similar ranking of the tested compounds between both *in vitro* iPSC-hBBB and primary rat models, we noticed species differences considering their interactions with ABC transporters.

## Results

### iPSCs generation and human brain endothelial cells differentiation

**Figure 1:**
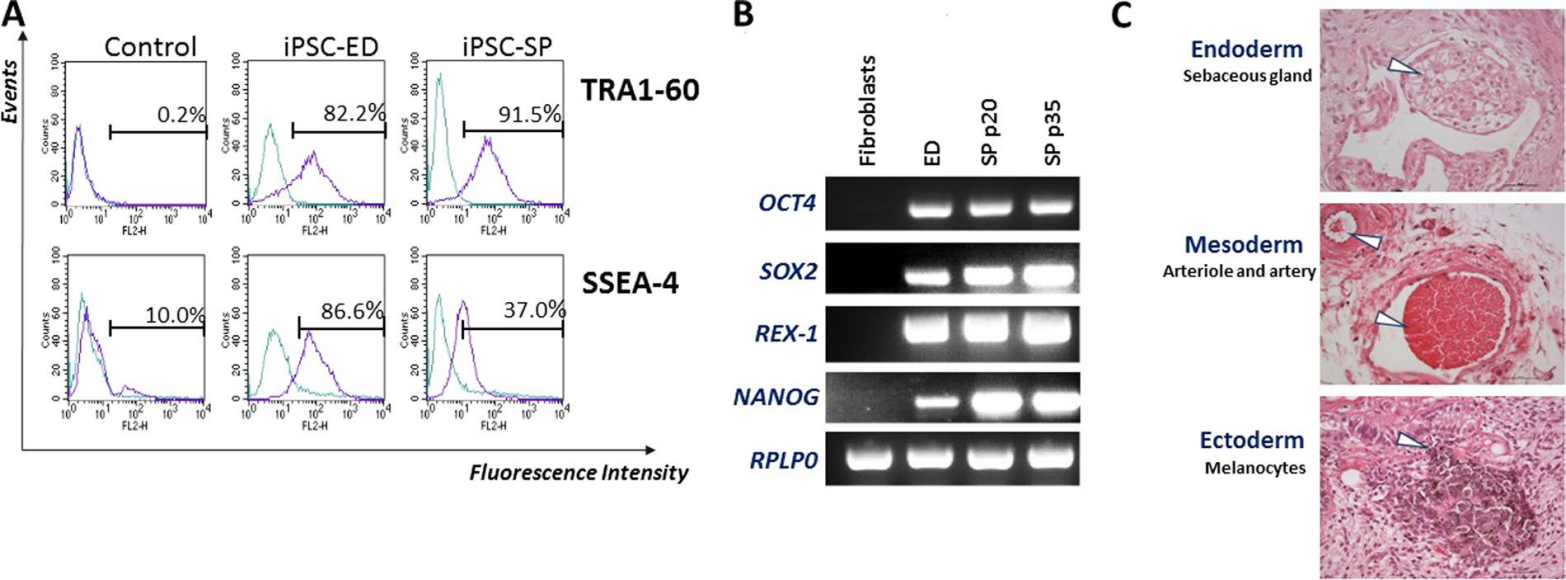
Establishment and characterization of iPSCs. (**A**) Representative analysis of TRA1-60 and SSEA-4 expression for each iPSC cell line by flow cytometry. TRA1-60 is compared with unlabeled cells and SSE-A4 is compared with the appropriate mouse IgG3 control. Control: green line; test: purple line. Samples of somatic control cells (Caco-2 epithelial cells for TRA1-60 and hBMEC for SSEA-4) were tested in parallel (respectively). Flow cytometry analysis of the iPSCs lines were performed in exponential growth phase. (**B**) Gene expression analysis of pluripotency markers in both iPSC and in fibroblast control by semi-quantitative PCR. (**C**) Ability of the iPSC-SP cell line to generate all three germ cell layer components within teratomas (endoderm – line 1, mesoderm – line 2 and ectoderm – line 3) was evaluated. Whole sections of teratoma were performed after 5 to 10 weeks of growth and stained with hematoxylin and eosin. Scale bare = 50 µm.

The ED-iPSC line has been provided through the Harvard Stem Cell Institute iPS Core Facility. For the SP-iPSC line, Placenta from fibroblasts explants (Serena Pavoni, PhD, CEA Fontenay aux Roses/SupBiotech, France) was reprogrammed using the Sendai virus method until colonies began to adopt an iPSC-like morphology 15 days after transduction with OCT4, KLF4, SOX2 and C-myc (data not shown). After 6 to 10 passages on mouse embryonic fibroblasts (MEFs), iPSC lines were adapted to feeder-free conditions (Matrigel) and regularly verified for their pluripotency state. iPSC lines expressed TRA-1–60 and SSEA4 markers (Fig. 1A). We showed the expression of the endogenous pluripotency marker genes *OCT4*, *SOX2*, *REX1* and *NANOG* (Fig. 1B) while no expression was shown for those genes in the primary cells. In addition, the iPSC lines were able to generate *in vivo* the three embryonic germ layers (ectoderm, endoderm and mesoderm; Fig. 1C).

### iPSC-derived human brain endothelial cells (iPSC-hBECs)

**Figure 2:**
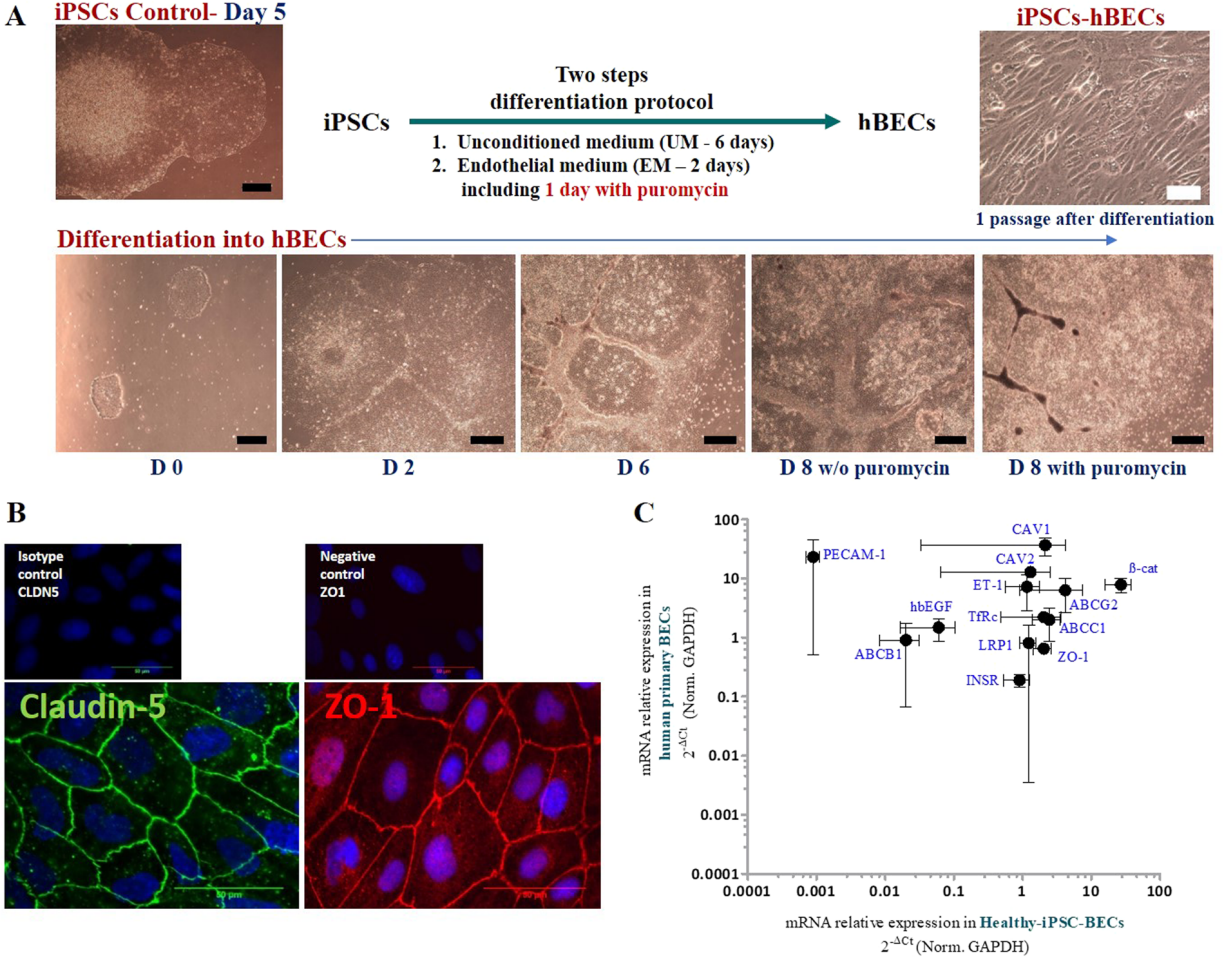
Differentiation of iPSC-hBECs. (**A**) Representative phase contrast images of iPSCs in cell culture at day 5 and after differentiation. By day 8, cells exhibited brain endothelial cell morphology in the presence of puromycin. Black scale bare, 500 µm. White scale bare, 50 µm. **(B)** ZO-1 and claudin-5 fluorescence intensity demonstrated that the differentiated cells exhibited brain endothelial characteristics. Scale bare, 50 µm. (**C**) Typical profile of transporter/receptor gene expression. The level of the expression of mRNA of junctional proteins like claudin-5 and ZO-1, transporters like ABCC1, ABCG2 and LRP1 in iPSC-Human BECs was comparable to that observed in our two primary hBECs samples.

Representative morphology changes of iPSC during differentiation are shown in Fig. 2A. The two step differentiation protocol adapted from Lipmann *et al*.^15^ with introduction of a selection step with puromycin yielded cells which exhibited brain endothelial cell morphology (Fig. 2A). Endothelial cells emerged at day 2 after addition of endothelial medium. The iPSC-hBECs showed morphology similar to that of primary cultures of brain endothelial cells with monolayers of tightly packed cells expressing ZO-1 and claudin-5 (Fig. 2B). In contrast to CD31 and ABCB1 mRNA, the mRNA expression level of transporters like ABCC1, ABCG2, TfRc and INSR in iPSC-hBECs was comparable to that observed in our primary brain endothelial cells (Fig. 2C).

### Functional and clinical validation of iPSC-hBBB models

**Figure 3:**
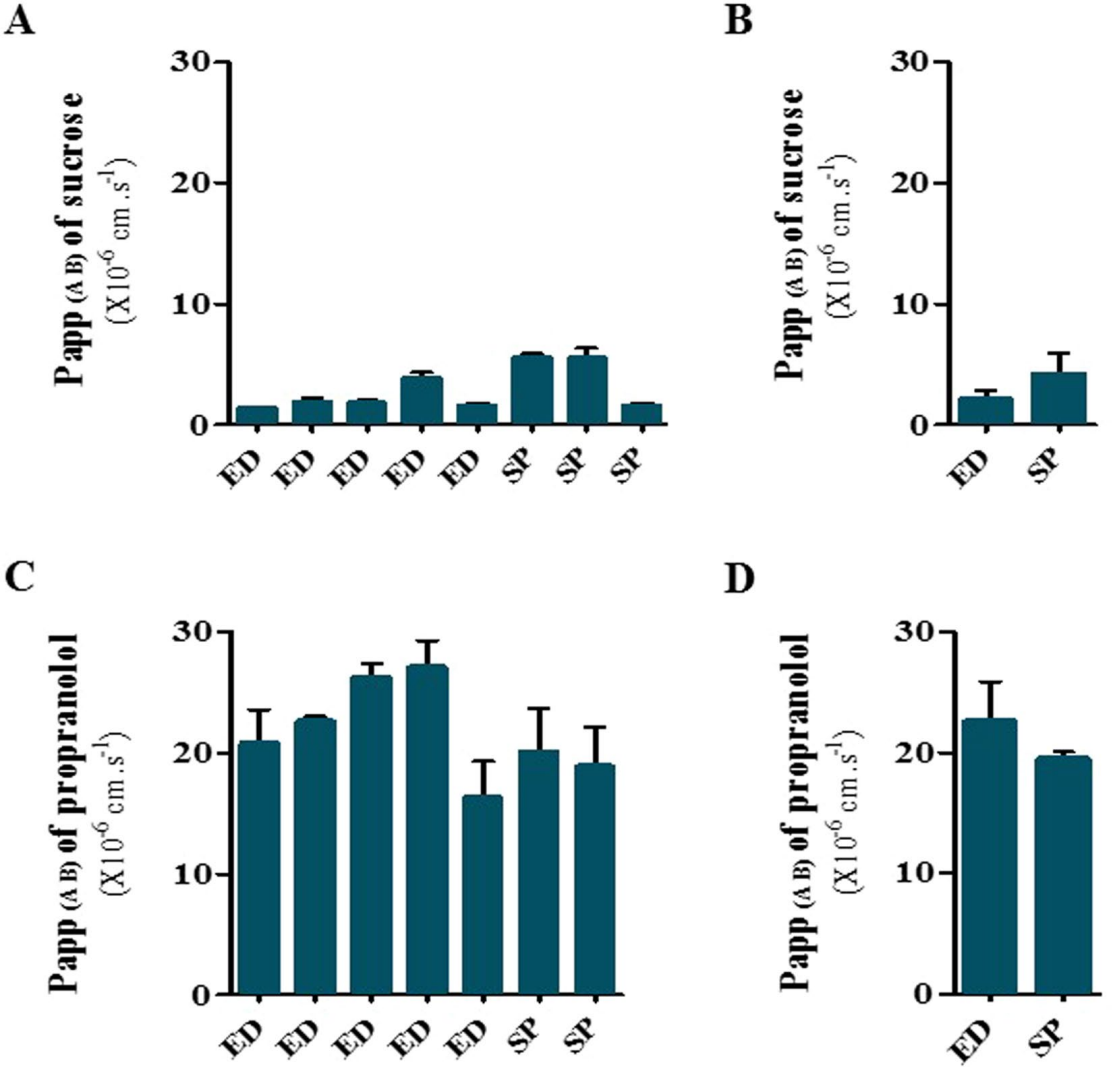
Low paracellular transport of sucrose and high permeability of propranolol in the *in vitro* iPSC-based human BBB model. (**A**,**C**) Each column represents the average of the apparent permeability (mean ± SD) from apical to basal compartment obtained on independent iPSC-BBB *in vitro* models (7 to 8 experiments). (**B**,**D**) each column represents the average of the apparent permeability from apical to basal compartment obtained in the two cell lines (mean ± SEM).

To further demonstrate that the *in vitro* iPSC-hBBB monolayer displayed well-developed tight junctions, we investigated the permeability of a paracellular marker, i.e. sucrose, across the brain endothelial cell monolayer. The mean permeability value of sucrose across the iPSC-hBBB monolayer generated with the two iPSC lines was 2.9 ± 1.6 × 10^−6^ cm.s^−1^ (Fig. 3B) in comparison with propranolol, a highly permeable BBB marker [21.7 ± 3.1 × 10^−6^ cm.s^−1^ (Fig. 3D)], indicating an overall restrictive paracellular permeation as confirmed by the average of TEER values (458 ± 225 Ω.cm^2^).

**Figure 4:**
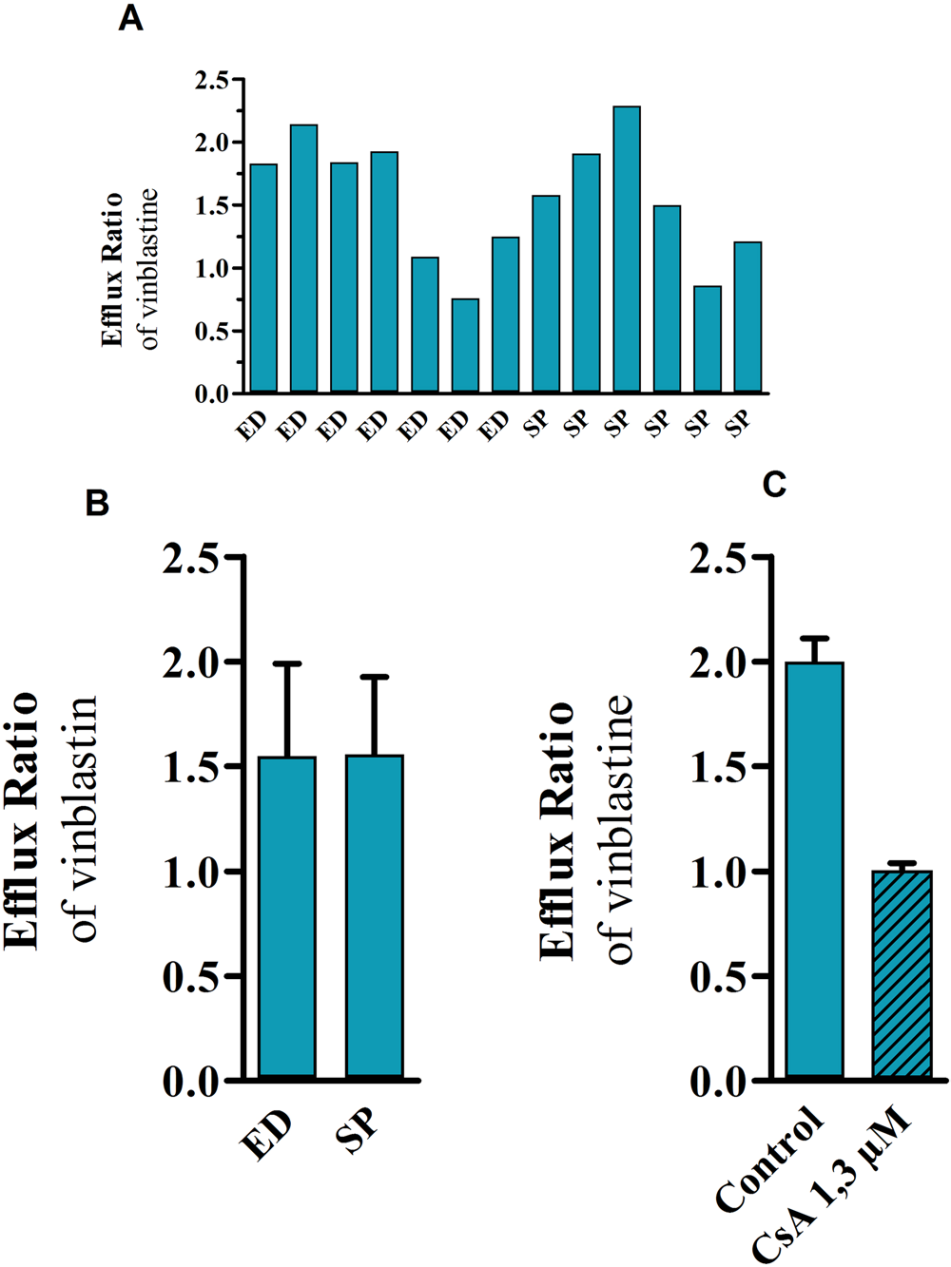
Assessment of the bidirectional transport of the P-gp transporter substrate vinblastine in the iPSC-hBBB models. (**A**) ER of iPSC-hBBB models from different iPSC batches. (**B**) ER mean ratio in iPSC-hBBB models from at least 6 identical iPSC showing the ER intra-variability (mean ± SEM). **(C**) Effect of CsA on the vinblastine ER (mean ± SD).

A known P-gp substrate was used to assess the asymmetry index of the iPSC- hBBB model. In this context, the apparent permeability of vinblastine was measured bi-directionally: from apical to basal (P_appAB_) and from basal to apical compartments (P_appBA_) in the presence or absence of the P-gp inhibitor cyclosporine A. The P_appBA_/P_appAB_ efflux ratio (ER) was calculated. The ER generated for each single batch of iPSCs is plotted in Fig. 4A; the mean value for the two iPSC- hBBB models from 13 independent experiments was 1.55 ± 0.40 [(CV: 25.80%); Fig. 4B]. P-gp inhibition using Cyclosporine A (CsA) resulted in a 50% decrease of vinblastine ER indicating the presence of functional P-gp efflux in the iPSC-hBBB model (Fig. 4C).

**Figure 5:**
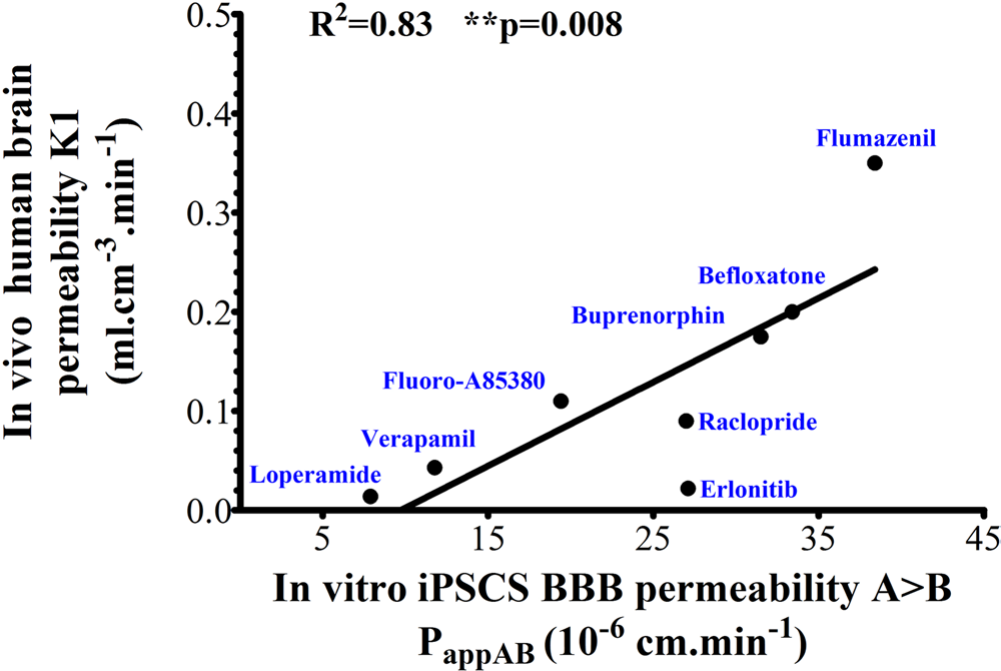
Clinical validation of iPSC- hBBB Blood-Brain Barrier model. Linear regression analysis shows a positive correlation between apparent permeability in human brain (*K*_1_) and *in vitro* apparent permeability in the iPSC-hBBB model. Spearman tests were performed and *P < 0.05 indicates y significant correlation.

**Figure 6:**
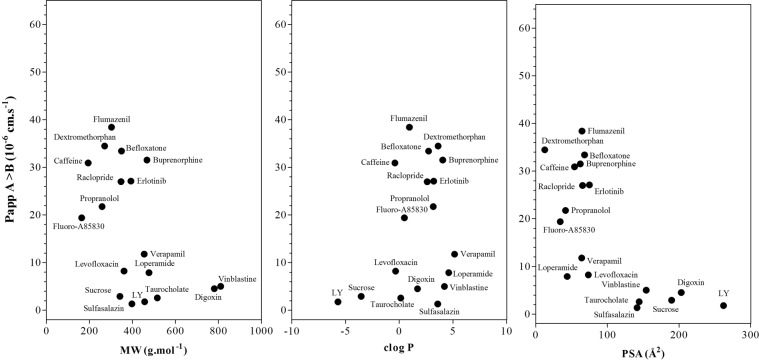
iPSC- hBBB Blood-Brain Barrier model permeability characterization. Apparent permeability values of marketed CNS and non-CNS drugs from the *in vitro* iPSC-hBBB model and relationship with their molecular weight (MW), cLogP, and surface polar area (PSA). Data are the average of two or three cell monolayers for each tested compound.

To further assess the relevance of the *in vitro* iPSC-hBBB model, we used compounds with known passage across the human BBB *in vivo* [rate constant for transfer from arterial plasma into brain obtained from compartment modeling of PET data, *K*_1_, mL.cm^−3^.min^−1^ (S1 Table 1)]. These parameters were compared with the apparent permeability obtained using the *in vitro* iPSC-hBBB model (P_appAB_). Permeability determined with the *in vitro* iPSC-hBBB model showed a highly significant correlation with the *in vivo K*_1_ (R^2^ = 0.83; *P* = 0.008; Fig. 5). While fluoro-A85380 and raclopride showed a moderate cerebral uptake, befloxatone, buprenorphine as well as flumazenil exhibited a high cerebral uptake in human brain and also a high permeability across the *in vitro* system. In contrast, loperamide and verapamil exhibited low cerebral uptake with limited *K*_1_ *in vivo* as well as a low apparent permeability *in vitro* (Fig. 5). A discrepancy was observed with erlotinib which exhibited a low cerebral uptake in human brain but high permeability *in vitro*. This could be explain by the fact that the concentration of erlotinib used *in vitro* induced inhibition of P-gp/BCRP expressed in the *in vitro* iPSC-hBBB model. Although this study needs to be extended to a larger set of radioligands with known PK parameters in human brain, our data suggest that the *in vitro* iPSC-hBBB model will be useful in drug screening strategies. In this context, we evaluated the permeability of a larger panel of marketed drugs across the *in vitro* iPSC-hBBB model. A robust and validated LC-MS/MS method was used to evaluate the ability of the *in vitro* iPSC-hBBB model to discriminate between CNS and non CNS drugs (Table 1). The mean P_app_ value for the marketed non-CNS compounds from the apical to basal compartment was about 2.1 ± 0.6 × 10^−6^ cm.s^−1^ (range: 1.3 × 10^−6^ cm.s^−1^ to 2.9 × 10^−6^ cm.s^−1^) which is consistent with a limited passage across the cell monolayer. With the exception of erlotinib, drugs known to be substrate of efflux transporter such as digoxin, loperamide, fluoro-A85380, verapamil and vinblastine are likely to be also ABC transporter substrates in the iPSC-hBBB model. The mean P_app_ value for CNS compounds such as dextromethorphan, caffeine, flumazenil, raclopride, buprenorphine, befloxatone, and propranolol, was about 30.1 ± 4 × 10^−6^ cm.s^−1^ (range: 21.7 × 10^−6^ cm.s^−1^ to 38.4 × 10^−6^ cm.s^−1^) consistent with high permeability (Fig. 5). Consistent with previous observations, no relationship between drug physicochemical properties and *in vitro* apparent permeability was evidenced despite the fact that compounds cLogP < 5, MW < 450 Da and PSA < 120 Å displayed high *in vitro* apparent permeability (Fig. 6).

**Table 1:** Summary of Physicochemical Properties of the Set of Selected Compounds.

Compounds	MW	cLogP	PSA
[^14^C]Sucrose	342	−3.55	189.5
2F-A85380-tartrate	164	0.48	34.1
[^3^H]Digoxin	781	1.70	203.1
[^3^H]Propranolol	259	3.16	41.5
[^3^H]Vinblastine	811	4.20	154.0
Befloxatone	349	2.73	68.0
Buprenorphine	468	4.04	62.2
Caffeine	194	−0.40	54.0
Dextromethorphan	271	3.62	12.5
Erlotinib	393	3.20	74.7
Flumazenil	303	0.94	64.4
Levofloxacin	361	−0.34	73.3
Loperamide	477	4.60	43.8
Lucifer Yellow	457	−5.70	262.0
Raclopride	347	2.60	65.3
Sulfasalazine	398	3.58	141.3
Taurocholate	516	0.13	144.2
[^3^H]Verapamil	455	5.14	64.0

### Species differences study

**Figure 7:**
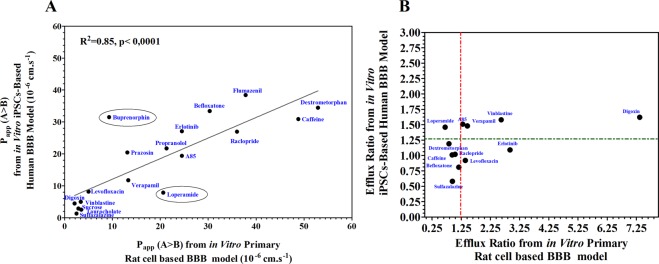
Species differences in blood-brain barrier transport. (**A**) Species correlation of apparent permeability in blood-brain barrier transport. (**B**) Species correlation of efflux ratio between *in vitro* primary rat cell based BBB model and *in vitro* iPSC-hBBB model. The dotted lines represent limits of ER.

We previously described an *in vitro* primary cell-based rat BBB model with relevant features of the *in vivo* rat BBB^6^. We therefore analyzed correlatively the permeability of a set of compounds obtained using the *in vitro* iPSC-hBBB model and the *in vitro* cell based rat BBB model. A good correlation was observed between the two models (R^2^ = 0.85; *P* < 0.0001). We noticed that the permeability value for compounds such as dextromethorphan, caffeine and loperamide was higher using the *in vitro* cell based rat BBB model than with the iPSC-hBBB model (Fig. 7A), while buprenorphine showed high permeability in only the iPSC-hBBB model. Fluoro-A85380, verapamil, vinblastine and digoxin were likely to be ABC-transporter substrates (ER ≥ 1.25) in both *in vitro* BBB models (Fig. 7B). However, loperamide apparently interacted with ABC transporters in the iPSC-hBBB but not in the *in vitro* cell based rat BBB model. Erlotinib was apparently an ABC transporter substrate in the primary rat model but not in the *in vitro* iPSC-hBBB model (Fig. 7B). These observations confirmed interspecies differences as has been pointed previously^6,21–23^.

## Discussions and Conclusions

We provide evidence of the physiological relevance of the iPSC-hBBB model in predicting human brain permeability to drugs by means of evaluation of the BBB permeability of radioligands studied correlatively *in vivo* with quantitative PET imaging in humans and *in vitro*. In addition, we report a lack of relationship between drug physicochemical properties and *in vitro* apparent permeability using the iPSC-hBBB model. Finally, we demonstrate interspecies differences in the prediction of drug permeability between the rodent BBB model and the iPSC-hBBB model in the prediction of drug BBB permeabilities. This is to our knowledge the first proof-of-concept study to compare transport results obtained using the iPSC-hBBB model and clinical data obtained using i*n vivo* PET imaging.

The *in vivo* measurement of BBB permeation is expensive and time-consuming. Moreover, a reduction in animal experiments is desirable on ethical grounds. In addition, species differences in BBB permeability between rodent and human brain justify the development of *in vitro* human BBB models to avoid failure in late clinical development, when upward of $100 million is typically invested in a drug candidate. However, the integration of these human BBB models in research screening flow schemes requires knowledge of their *in vivo* BBB predictivity in brain permeation of drug candidates. Many *in vitro* human BBB models generated from iPSCs have been described recently^11–20^. The major hurdle associated with these models is a lack of a cross correlation between *in vitro* and *in vivo* pharmacokinetic data in demonstrating the predictive power of drug screening tools.

In an effort to achieve an *in vivo* like BBB phenotype, Lipmann’s protocol for the differentiation of iPSCs into BECs was modified by introducing a puromycin selection step. By doing so we produced well differentiated BECs as shown by localized expression of Claudin-5 and Zo-1, the integral membrane components of tight junctions. This overall expression of tight junction proteins in iPSC-BECs was associated with restricted permeability to the paracellular permeability marker sucrose in the iPSC-hBBB model and confirmed by the average (n = 13 independent experiments) of TEER value (458 ± 225 Ω.cm^2^**)**. The mean P_app_ value of sucrose in the *in vitro* iPSC-hBBB model from eight different batches of cells in at least two replicates was 2.9 ± 1.6 × 10^−6^cm.s^−1^. The TEER values obtained in our experimental system without retinoic acid cell activation are in the same range as the current TEER standards^16,19^. In addition, our model displayed a differential permeability for diverse CNS and non CNS drugs. However, no relationship was evidenced between drug permeability and physicochemical properties of the compound such as PSA (R^2^ = −0.65), MW (R^2^ = −0.52), and lipophilicity (R^2^ = 0.20). The *in vitro* iPSC-hBBB model displayed a dynamic range, with P_app_ values ranging from 1.3 to 38.4 × 10^−6^ cm.s^−1^ for the CNS and non CNS drugs. Our data are in agreement with our previous observation using *in vitro* primary human model^6^.

Since the predictivity of human brain permeability of drug candidates by the recently developed *in vitro* iPSC-hBBB model has not yet been demonstrated, we compared previously published *in vivo* brain pharmacokinetic data of selected radioligands with their *in vitro* permeability using our iPSC-hBBB model. *In vivo* pharmacokinetic data were obtained with PET imaging, which is a powerful tool to assess BBB penetration of drugs *in vivo* in humans^24^. Brain kinetics can be analyzed by compartmental modelling, which allows calculation of the relevant rate constants that describe BBB passage. Although our study needs to be extended to a large set of radioligands with known BBB permeation in human brain, Fig. 5 reveals good agreement between the *in vitro* and *in vivo* human brain pharmacokinetic data (R^2^ = 0.83, *P* = 0.008), suggesting the predictive value of the *in vitro* iPSC-human BBB model in the evaluation of the brain penetration of drug candidates. While raclopride and fluoro-A85380 exhibited a moderate brain distribution, Fig. 5 reveals a low *in vivo* brain distribution in humans for loperamide and verapamil in comparison with befloxatone, buprenorphine and flumazenil. The transfer rate constant from plasma into brain (*K*_1_) and the distribution volume (*V*_T_) of these compounds was shown to be dependent on their interaction with efflux transporters, such as P-gp, as previously reported^7,25–27^. Discrepancies could be observed between the brain permeation of ^11^C-erlotinib in humans and the permeation of erlotinib across the iPSC-hBBB model. Saturation of the carrier-mediated efflux of erlotinib at the BBB has been reported *in vivo* in nonhuman primates using high doses of erlotinib^28^. It may be hypothesized that the tested concentration of erlotinib might inhibit its own transport by P-gp and BCRP across iPSC-hBBB in our experimental setting. The functional expression of P-gp was demonstrated by the use of the P-gp substrate vinblastine. The ER for this drug from 13 independent experiments was about 1.55 ± 0.40. This efflux was inhibited by cyclosporine-A suggesting the presence of functional P-gp efflux in the iPSC-hBBB model. We confirmed that the level of mRNA expression of ABCG2 and ABCC1 in iPSC-BECs was higher than for ABCB1. These results are consistent with previous observations showing that the abundance of BCRP is higher in human brain microvessels than that of P-gp^29–31^. In addition, Fig. 2C reveals mRNA expression of insulin and transferrin receptors in the same range as for primary human BECs, while the mRNA expression of caveoline 1 and caveoline 2 is 10 times lower than in BECs.

To summarize, the iPSC-hBBB model as described in this report mimics *in vivo* BBB integrity and compound permeability. Collectively, our findings suggest that the *in vitro* iPSC-hBBB model could be integrated in a flow chart screening of promising compounds for the treatment of brain diseases. In this view, miniaturization strategies will be developed for set up the models and for high throughput screening.

## Materials and Methods

### Drugs selection

Eight compounds with known pharmacokinetic parameters in human brain were selected: befloxatone (Synthélabo), flumazenil (Sigma), raclopride (tartrate salt, Sigma), erlotinib (Fluorochem), verapamil (Sigma Aldrich), buprenorphine (Sigma Aldrich), 2F-A85380-tartrate (Eras Labo) and loperamide (Sigma). In addition, eleven other compounds with diverse physicochemical properties were selected. We tested six non-radiolabeled compounds: dextromethorphan (hydrobromide monohydrate, Sigma Aldrich), levofloxacin (Sigma), sulfasalazine (Sigma), caffeine (Sigma Aldrich), taurocholate (sodium salt, Sigma), Lucifer Yellow (CH dilithium salt, Sigma); and five radiolabeled compounds: [U−^14^C]-sucrose (molar activity: 601 mCi.mmol^−1^), [^3^H]propranolol (molar activity: 24 Ci.mmol^−1^), [^3^H]verapamil hydrochloride (molar activity: 85 Ci.mmol^−1^), [^3^H]vinblastine sulfate (molar activity: 10.3 Ci.mmol^−1^), [^3^H]digoxin (molar activity: 40.0 Ci.mmol^−1^) were purchased from Perkin Elmer (Massachusetts, SA).

### Animal use and assurances

Animal experiments were conducted in full compliance with local, national, ethical, and regulatory principles and local licensing regulations, per the spirit of the Association for Assessment and Accreditation of Laboratory Animal Care. The animal studies were carried out in accordance with decree 87–848, issued on October 19, 1987, concerning animal experimentation in France, in an approved facility (accreditation No. A91505) and by workers with Ministry of Agriculture authorization, as specified in the decree. The experimental protocol was approved by the Ethic committee for animal experiments, Life Sciences Division, Fundamental Research Department, CEA Fontenay aux Roses, France).

### Human brain endothelial cells and derivation of patient-fibroblasts

Human brain endothelial cells used in this study were obtained from adult brain tissue after agreement of local ethics committee (Biomedical Agency-PFS09). All procedures were carried out in accordance with relevant guidelines and regulations. The informed consent was obtained from the legal guardian. BECs were isolated as described previously^6^.

iPSCs-SP derived from ifbroblast cells were a generous gift of SEPIA’s Laboratory (CEA Fontenay aux Roses).

Fibroblast cells were isolated from normal placenta according to the protocol study approved by the local ethics committee (Advisory Committee for the Protection of Persons in Biomedical Research Cochin Hospital, Paris, n°18-05). All procedures were carried out in accordance with relevant guidelines and regulations. The human samples were obtained with a written and informed consent, which was supervised and anonymized.

After 1–2 weeks, fibroblasts outgrowths from the explants were passaged and maintained as described previously^32^ in Dulbecco’s Modified Eagle Medium (DMEM, Life Technologies, Carlsbad, CA) supplemented with 10% inactivated fetal bovine serum (FBS, Life Technologies), 1% penicillin/streptomycin/neomycin (PSN, Life Technologies), 1% sodium pyruvate (Sigma-Aldrich, St. Louis, MO), and 1% L-glutamine (Life Technologies).

### iPSC-Generation and Characterization

#### Reprogramming

Fibroblasts were reprogrammed using the Sendai virus reprogramming method as recommended by the manufacturer (Life Technologies). Briefly, fibroblasts were infected using SeV vectors encoding OCT3/4, SOX2, KLF4, and c-MYC on day 0. Two days later, cells were trypsinized and picked up onto two 10-cm gelatin-coated culture dishes that had been seeded with irradiated mouse embryonic fibroblasts (irrMEFs, GlobalStem). The cultures were maintained in human embryonic stem cell (hESC) medium containing DMEM/F12 (Gibco), 20% KnockOut Serum Replacement (Gibco), 1% minimum essential medium (MEM) nonessential amino acid (Gibco), 1 mM l-glutamine (Gibco), 0.1 mM β-mercaptoethanol (Sigma-Aldrich), and 10 ng/mL of basic fibroblast growth factor (β-FGF, Stem Cell technologies). Clones were picked starting on 20 days post infection, and expanded on irrMEFs before being adapted to feeder-free conditions.

#### hiPSC culturing

All iPSCs cell lines were maintained on hESC-qualified Matrigel (Corning®, NY, USA) in mTeSR^TM^1 medium (STEM CELL Technologies, Vancouver, BC, Canada) and passaged using mechanical dissociation at the beginning of the establishment of the cell line. After 7 to 10 passages, cells where passaged using ReLeSR^TM^ (STEM CELL Technologies) and maintained until the passage 40 for the experiments.

#### Teratoma formation

To examine the developmental potential of reprogrammed clones *in vivo*, iPSCs grown on feeder free system, were collected by dispase treatment, and injected into hind limb muscle of 8-week-old immunodeficient NOD-scid IL2rγnull (NSG) mice (approximately 1 × 6-well plate at 70% confluence for each injection). After five to ten weeks, teratomas were dissected and fixed in 4% paraformaldehyde. Samples were embedded in paraffin and processed with hematoxylin and eosin staining. All studies were done with compliance with animal welfare regulations.

#### Assessment of pluripotency

The pluripotency characteristics of iPSC was demonstrated (i) by assessing the presence of totipotence gene expression marker by RT-PCR and (ii) by measuring their expression by flow cytometry.

(i). Gene expression assessment. Total RNA was extracted from iPSCs using Nucleospin RNA II Kit (Macherey-Nagel) according to the manufacturer’s instructions. Reverse transcription (RT) was carried out using Iscript cDNA synthesis kit (Biorad) following the instructions provided by the manufacturer. A total of 1 µg of RNA was used in RT reactions. RT-PCR for the 5′ coding region was performed with primers specific for OCT4, SOX2, REX-1, NANOG and RPLO (housekeeping gene) (S2 Table 2).
(ii). Flow Cytometry analysis. hiPSCs were harvested by accutase treatment for 5 to 10 min until the cells detach. Cells were then fixed in either paraformaldehyde 4% for 8 min. or in ice-cold methanol for 15 min.After two PBS1X wash, cells were blocked with 40% of goat serum in PBS1X for 20 min at room temperature. After 2 wash in 10% of goat serum in PBS1X (10%PBSG), cells were incubated for 1 h at room temperature or overnight at 4 °C with primary antibodies diluted in 10%PBSG. IgG controls were used at the same concentration. Cells were then washed twice in 1.5 ml of PBS1X-5%SVF and resuspended in 500 µL of FACS Flow for analysis on a FACS calibur flow cytometer. References and working dilutions of antibodies are plotted in S3 Table 3.

### Derivation of human brain endothelial cells from iPSCs (hiPSC-BECs)

Human iPSCs were differentiated as described previously^15^ with substantial modifications. Briefly, cells were passaged using ReLeSR^TM^ onto Matrigel and maintained in mTeSR1 medium for 2–3 days. After expansion, cells were switched on unconditioned medium (DMEM/Ham’s F-12, GlutaMAX^**TM**^ supplement (Life Technologies) containing 20% KnockOut Serum Replacement (Life Technologies), 1% MEM Non-Essential Amino-Acid Solution (Life Technologies) and 0.055 mM β-mercapto-ethanol (Life Technologies)) for 6 days. Cells were cultivated for two additional days in endothelial medium (Human Serum Free Medium (hSFM, Gibco) supplemented with 20 ng/mL β-FGF (Life Technologies) and 1% of platelet-poor plasma derived bovine serum (AlfaAesar, Ward Hill, USA)). To improve the selection of endothelial cells, 1 µg/mL of puromycin (Sigma) was added during the first day of endothelial medium step. At day 8, cells were then gently dissociated with Accumax (Sigma) for 5–8 min at 37 °C and plated onto 24-well tissues culture polystyrenes plates or 1.12 cm^2^ Transwell^®^ permeable inserts (0.4 µm pore size) coated firstly for 2 hours with collagen IV 100 µg/mL (Sigma) and then for 1 hour with fibronectin 10 µg/ml (Sigma). Endothelial cells were then grown in endothelial medium for co-culture experiments or immunohistochemistry.

### Transcription profiles of tight junctions, efflux transporters and other receptors

To evaluate quality of differentiation of hiPSCs-BECs, the mRNA expression profile of specific endothelial markers was assessed. RNA was isolated from hiPSC-BECs or iPSCs using the RNeasy Mini kit (Qiagen), according to the manufacturer’s instructions. The concentration and purity of RNA samples were checked spectrophotometrically at 260 and 280 nm using the NanoDrop ND-1000 instrument (NanoDrop Technologies, Wilmington, DE, USA); the A260/280 ratio ranged from 1.8 to 2. A sample of 0.5 μg of total RNA was converted to cDNA with random primers in a volume of 20 μL using an RT^2^ HT first stand kit (Qiagen) according to the manufacturer’s protocol. cDNA was diluted with water to a final volume of 200 μL. Quantitative expression of markers was determined using 2 µL of diluted cDNA for each primer (S4 Table 4) set at 10 µM using iQ SYBR Green Supermix (Biorad) in a final volume of 12 µL. Thermocycling was carried out in CFX96 RT-PCR detection system (Bio-Rad) using SYBR green fluorescence detection. The specific amplification conditions were 10 min at 95 °C, followed by 40 amplification cycles at 95 °C for 15 s, 60 °C for 60 s and 72 °C for 30 s to reinitialize the cycle again. The specificity of each reaction was also assessed by melting curve analysis to ensure the presence of only one product. Relative gene expression values were calculated as 2^−ΔCT^, where ΔCT is the difference between the cycle threshold (CT) values for genes of interest and housekeeping gene (GAPDH).

### iPSC-derived human BBB model

On day before the co-culture establishment, rat glial cells (2 × 10^4^ cells) isolated as described previously^6^ were plated on 12 wells plate (Costar) in glial specific medium. After 24 hours, hiPSC-BECs (6 × 10^4^cells) were plated on the upper side of a collagen-coated polyester Transwell membrane (Costar, pore size 0.4 µm; diameter 12 mm; insert growth area 1 cm^2^) in 0.5 mL of endothelial medium. Under those conditions, the hiPSC-BECs monolayer were confluent after 4 to 7 days of co-culture. The integrity of the cell monolayer was demonstrated (i) by assessing the Transendothelial Electrical Resistance (TEER), (ii) the assessment of presence of tight junctions between the endothelial cells by immunocytochemistry, (iii) by measuring the flux of the paracellular reference marker, [^14^C]sucrose, through the monolayer and (iv) by assessment of hiPSC-BECs polarity by measuring the efflux of P-gp substrates, i.e. vinblastine.

For TEER, cell culture inserts were transferred to an Endohm-6 chamber (Word Precision Instruments, Sarasota, FL) containing endothelial medium pre-warmed at 37 °C and resistance of the monolayer was measured using an EVOM2 Epithelial Volt Meter (World Precision Instruments). iPSC-hBBB models were considered mature when TEER measurements were stable and reached at least a mean of 170 ohm/cm^2^.

For immunocytochemistry, hiPSC-BECs monolayers were washed once in PBS1X and fixed in 4% paraformaldehyde for 8 min. After washing with PBS1X, cells were then soaked in a blocking solution containing 5% of BSA. Primary antibodies and isotype controls were diluted in the same solution and incubated on the monolayer for 1 hour at room temperature or overnight at 4 °C. After three washes with PBS1X, the cells were incubated for 30 min in the presence of secondary antibodies: goat anti-rabbit Alexa Fluor 594 (1:500; Invitrogen) or goat anti-mouse Alexa Fluor 388 (1:500; Invitrogen) diluted in blocking solution. References and working dilutions of antibodies are summarized in S2 Table 2. Cell nuclei labelling was performed at 1400 nM 4′,6-Diamidino-2-pheny-lindoldihydrochloride (DAPI) for 10 min and then washed 3 times in PBS1X. The preparations were observed with a fluorescence microscope and pictures were processed on ImageJ (NIH).

The integrity of iPSC-derived human BBB model was determined by assessment of radiolabelled sucrose permeability as a paracellular marker. The transwells with hiPSC-BECs monolayers were transferred to new 12 well plates. Transfer buffer (150 mM NaCl, 5.2 mM KCl, 2.2 mM CaCl_2_, 0.2 mM MgCl_2_, 6 mM NaHCO_3_, 2.8 mM glucose and 5 mM Hepes) pre-warmed at 37 °C was added: 1.5 mL to the basolateral compartment (B) and 0.5 mL to the apical compartment (A) containing 0.1µCi/mL (166-300 nM) of [^14^C]sucrose (Perkin Elmer). After 1 hour of incubation at 37 °C with gentle rotation, 100 µL of transfer buffer from both A and B compartments were collected and counted by scintillation. The P_app_ value was calculated as followed:

$${{\rm{P}}}_{{\rm{app}}}={\rm{dQ}}/{\rm{dT}}\,{\rm{X}}\,{\rm{A}}\,{\rm{X}}\,{{\rm{C}}}_{{\rm{0}}}$$

where dQ/dT is the amount of compound transported per time point; A is the membrane surface area of the insert; and Co is the donor concentration at the time point. Data were presented as the average ± SD from three monolayers. Monolayers were validated for sucrose permeability from A to B when the P_app_ was below 8 × 10^−6^ cm.s^−1^. The propranolol permeability was also tested using the same protocol with an apical tested dose about 1 µCi/mL (48 nM) of [^3^H]propranolol in transfer buffer. Monolayers were validated for propranolol permeability from A to B when the P_app_ was under 16 × 10^−6^ cm.s^−1^. For the activity of efflux pumps and the asymmetry of transporters distribution of the endothelial cells, we measured the efflux of [^3^H]vinblastine or [^3^H]verapamil across the cell monolayer. 0.1 µCi.mL^−1^ of [^3^H]vinbastine (4.8-9.7 nM) or [^3^H]verapamil (1.25 nM) were applied in the apical or basal compartment and the P_app_ for the passage of compartment A to B (P_app A>B_) and B to A (P_app B>A_) were calculated. The efflux ratio (ER) was calculated as follows: ER = P_app B>A_/P_app A>_B_._ The specificity of this efflux was confirmed by inhibition of vinblastine efflux with the P-gp inhibitor cyclosporine-A at low concentration (1.3 µM).

### Permeability studies

After checking the cell monolayer integrity, the monolayers were transferred to new plates. The buffer (150 mM NaCl, 5.2 mM KCl, 2.2 mM CaCl2, 0.2 mM MgCl2, 6 mM NaHCO3, 2.8 mM glucose and 5 mM Hepes) was added: 1.5 mL to the basal chamber (B) and 0.5 mL to the apical chamber (A). Experiments were performed in triplicate for each compound. The tested compounds (5–10 μM) were introduced in the donor chamber (either the apical or the basal compartment). After 60 min, aliquots were removed from the acceptor and basal chambers for drug-concentration determination by LC-MS/MS.

For LC-MS/MS a sample aliquot (transfer buffer) was diluted by half with acetonitrile and then, 10 μL of this solution was injected into the analytical equipment made up a Liquid Chromatography system coupled to a triple quadrupole mass spectrometer Thermofisher TS Quantum Discovery or Waters Quattro Premier XE equipped with a electrospray ionization source. System control and data processing were carried out using Xcalibur or Masslynx software. The chromatographic separation was performed on a Waters ACQUITY UPLC BEH C18 column (2.1 mm × 50 mm, 1.7 μm) at 60 °C. A gradient of two solvent mixtures was delivered at 0.3 mL/min or 0.5 mL/min according to the analytical system. Solvent A comprised 0.1% formic acid in ammonium acetate (5 mM) and solvent B, 0.1% formic acid in acetonitrile. Molecules were ionized in positive mode. Multiple reaction monitoring mode was used for compounds quantification. For all tested compounds, a linear gradient of mobile phase A and B was optimized. Linearity of the response was verified in the range 0.05 to 5 µM through 3 experiments to be able to measure low passage. Quantification was carried out using standard calibration curve and quality control samples prepared with blank matrix (transfer buffer/acetonitrile; 1/1; v/v) spiked with compounds.

### Statistical analysis

Statistical analysis was performed using the GraphPad Prism 5.04 program (GraphPad Software, Inc., San Diego, CA). Regression lines were calculated and correlation was estimated a nonparametric Spearman test. Statistical significance was set at p < 0.05.

## Supplementary information

Supplementary informations
