# Supplementary material for: Proof-of-Concept Study of Drug Brain Permeability Between in Vivo Human Brain and an in Vitro iPSCs-Human Blood-Brain Barrier Model
Source: Sci Rep. 2019 Nov 5;9:16310. doi: 10.1038/s41598-019-52213-6 (PMC6831611; doi:10.1038/s41598-019-52213-6)
Supplement: Supplementary file 1 — Supplementary informations [file 41598_2019_52213_MOESM1_ESM.pdf]

SUPPLEMENTAL INFORMATIONS

Proof-of-Concept Study of Drug Brain Permeability Between *in Vivo* Human Brain and an *in Vitro* iPSCs-Human Blood-Brain Barrier Model.

Gwenaëlle Le Roux[1], Rafika Jarray [2,3], Anne-Cécile Guyot[1], Serena Pavoni[2], Narciso Costa[1], Frédéric Théodoro[4], Ferid Nassor[2,3], Alain Pruvost[4], Nicolas Tournier[5], Yulia Kiyan[6], Oliver Langer[7], Frank Yates [2,3], Jean Philippe Deslys[2], Aloïse Mabondzo[1*]

[1]Service de Pharmacologie et d'Immunoanalyse, CEA, Université Paris-Saclay, F-91191 Gif-sur-Yvette, France.

[2]Service d'Etude des Prions et des Infections Atypiques, CEA, F-92265 Fontenay-aux-Roses, France.

[3]Sup'Biotech, F-94800 Villejuif, France.

[4]Service de Pharmacologie et d'Immunoanalyse (SPI), Plateforme Smart-MS, CEA, INRA, Université Paris-Saclay, F-91191 Gif-sur-Yvette, France.

[5]UMR 1023 IMIV, Service Hospitalier Frédéric Joliot, CEA, Inserm, Univ. Paris Sud, CNRS, Université Paris-Saclay, Orsay, France.

[6] Medizinische Hochschule Hannover, DE-30625 Hannover, Germany.

[7] Department of Clinical Pharmacology, Medical University of Vienna, A-1090 Vienna, Austria and Preclinical Molecular Imaging, AIT Austrian Institute of Technology GmbH, A-2444 Seibersdorf, Austria.

S1-Table 1. Rate constant for transfer from arterial plasma into brain obtained from compartimental modelling of PET data

**Clinical PET radioligands**	***K*1 (mL.cm^{-3}.min^{-1})**	**References**
**Befloxatone**	0.20	unpublished data
**Fluoro-A85380**	0.11	33
**Flumazenil**	0.35	34
**Raclopride**	0.09	35
**Loperamide**	0.002	27
**verapamil**	0.04	36
**Erlotinib**	0.02	37
**Buprenorphine**	0.17	Unpublished data

S2-Table 2. Primer sequences used for semi-quantitative PCR analysis

**Primer name**	**Primer sequence (5'>3')**
**OCT4_forward**	AGCGAACCAGTATCGAGAAC
**OCT4_reverse**	TTACAGAACCACACTCGGAC
**SOX2_forward**	AGCTACAGC ATGATGCAGGA
**SOX2_reverse**	GGTCATGGAGTTGTACTGCA
**REX1_forward**	CAGTCCAGCAGGTGTTTGC
**REX1_reverse**	GCATTCTATGTAACAGTCTGAGA
**NANOG_forward**	TGAA CCTCAGCTACAAACAG
**NANOG_reverse**	TGGTGGTAGGAAGAGTAAAG
**RPLPO_forward**	CATTGCCCCATGTGAAGTC

S3-Table 3. References and working dilutions of antibodies used for immunofluorescence and flow cytometry experiments.

Targeted antigen	Antibody description	References / supplier	Final concentration for IF or Flow cytometry
**ZO-1 (Clone ZMD.437)**	Rabbit polyclonal anti-human	40-2300 / Thermo Fisher Scientific	5 µg/ml (IF)
**Claudin-5 (Clone 4C3C2)**	Mouse monoclonal anti-human	35 250 / ZYMED	10 µg/ml (IF)
**Anti-mouse Alexa Fluor 488**	Polyclonal Goat anti-mouse	A-11029 / Thermo Fisher Scientific	5 µg/ml (IF)
**Anti-rabbit Alexa Fluor 594**	Polyclonal Goat anti-rabbit	A-11037 / Thermo Fisher Scientific	5 µg/ml (IF)
**SSEA4 (clone eBioMC-813-70)**	Monoclonal Mouse anti-human	12-8843-42 / eBioscience	20 µg/ml (Flow cytometry)
**PE Mouse IgG3 Isotype Control (Clone B10)**	Mouse IgG3	12-4742-42 / eBioscience	20 µg/ml (Flow cytometry)
**TRA1-60 PE (Clone TRA1-60)**	Monoclonal Mouse anti-human	12-8863-42 / eBioscience	20 µg/ml (Flow cytometry)

S4-Table 4. Primer sequences used for RT-Q-PCR analysis

Primer name	Primer sequence (5'->3')
**hCAV1_forward**	AGTTGCTGCAAACCTGACC
**hCAV1_reverse**	CTCCTCCCCCATCTTCTTTC
**hCAV2_forward**	CAGTGCAGACAATATGGAAGAG
**hCAV2_reverse**	GGAAATGAACAGAACAGTGGTG
**hβ-catenin_forward**	TCTTACACCCACCATCCCAC
**hβ-catenin_reverse**	GCACGAACAAGCAACTGAAC
**hEndothelin1-forward**	CTCCTGCTCGTCCCTGATG
**hEndothelin1-reverse**	CGGTCTGTTGCCTTTGTGG
**hTFRC_forward**	TCCCTTCCTTCAATCACACTC
**hTFRC_reverse**	TCTTTCAGCACATTGCTCAC
**hINSR-forward**	TGTTCATCCTCTGATTCTCTG
**hINSR-reverse**	GCTTAGATGTTCCCAAAGTC
**hbEGF-forward**	TGACCACACAACCATCCTG
**hbEGF-reverse**	TCCACATCATAACCTCCTCTC
**hPgp-forward**	TGAATCTGGAGGAAGACATGAC
**hPgp-reverse**	CCAGGCACCAAAATGAAACC
**hMRP1-forward**	AATAGAAGTGTTGGGCTGAG
**hMRP1-reverse**	CGAGACACCTTAAAGAACAG
**hBCRP1-forward**	TGCCCAGGACTCAATGCAAC
**hBCRP1-reverse**	ACGTGATTCTTCCACAAGCCC
**hLRP1-forward**	TGCTACTGCAACAGCAGCTTTC
**hLRP1-reverse**	TGCCGTACACTGAGCACTCATC

**hZO1-forward**	TGATCATTCCAGGCACTCG
**hZO1-reverse**	CTCTTCATCTCTACTCCGGAGACT
**hCD31-forward**	GAGTATTACTGCACAGCCTTCA
**hCD31-reverse**	AACCACTGCAATAAGTCCTTTC
**hGAPDH-forward**	TCAAGAAGGTGGTGAAGCAGGC
**hGAPDH-reverse**	AGTGGGTGTCGCTGTTGAAGTC

References

33. Gallezot, JD. *et al.* In vivo imaging of human cerebral nicotinic acetylcholine receptors with 2-18F-fluoro-A-85380 and PET. *J Nucl Med.* **46**, 240-247 (2005).

34. Delforge, J. *et al.* Quantification of benzodiazepine receptors in human brain using PET, [^{11}C]flumazenil, and a single-experiment protocol. *J Cereb Blood Flow Metab.* **15**, 284-300 (1995).

35. Pappata, S. et al. n vivo detection of striatal dopamine release during reward: a PET study with [^{11}C]raclopride and a single dynamic scan approach. *Neuroimage* **16**, 1015-1027 (2002).

36. Bauer, M. *et al*. Approaching complete inhibition of P-glycoprotein at the human blood-brain barrier: an (R)-[^{11}C]verapamil PET study. *J Cereb Blood Flow Metab.* **35**, 743-746 (2015).

37. Bauer, M. *et al*. A Proof-of-Concept Study to Inhibit ABCG2- and ABCB1-Mediated Efflux Transport at the Human Blood-Brain Barrier. *J Nucl Med.* ***60***, 486-491 (2019).
